# ASSESSING THE FACTORS ASSOCIATED WITH NURSING HOME CLOSURES

**DOI:** 10.1093/geroni/igac059.1316

**Published:** 2022-12-20

**Authors:** Kelly Hughes, Qinghua Li, Zhanlian Feng, Iara Oliveira, Judith Dey

**Affiliations:** RTI International, Research Triangle, North Carolina, United States; RTI International, Waltham, Massachusetts, United States; RTI International, Research Triangle Park, North Carolina, United States; Office of the Assistant Secretary for Planning and Evaluation, Washington, District of Columbia, United States; Office of the Assistant Secretary for Planning and Evaluation, Washington, District of Columbia, United States

## Abstract

The media has reported recent increases in nursing home closures. This study examined closures from 2011-2019, identified facility and market characteristics associated with closures, and assessed the impact of closures on quality and access. We identified closures using termination dates and gaps in certification surveys and conducted descriptive and multivariate analysis. We identified 1,220 closures, with large increases in closure rates in 2018 and 2019 and geographic clusters. Chain facilities, urban facilities and smaller facilities were more likely to close, as were facilities with higher percentages of non-white and Medicaid residents. Staffing and quality five-star ratings had a nonlinear relationship with closure, which suggests Medicaid funding may impact closures rates. We found both the number of beds per 1,000 elderly and occupancy rates decreased, including in high-quality facilities. Closures should be examined further in the context of the COVID-19 pandemic.

